# Two dimensional Airy beam soliton

**DOI:** 10.1038/s41598-022-12758-5

**Published:** 2022-05-31

**Authors:** Thomas Bouchet, Nicolas Marsal, Marc Sciamanna, Delphine Wolfersberger

**Affiliations:** 1 Chair in Photonics, CentraleSupélec, LMOPS, 2 Rue Edouard Belin, 57070 Metz, France; 2 Université de Lorraine, CentraleSupélec, LMOPS, 2 Rue Edouard Belin, 57070 Metz, France

**Keywords:** Nonlinear optics, Solitons

## Abstract

We demonstrate the formation of a two dimensional Airy beam soliton in a photorefractive crystal. By simply varying the nonlinearity strength we identify several scenarios showing the coexistence between an Airy beam and the emerging soliton. The soliton output profile behaves according to the theoretical soliton existence curve and can be tailored by the nonlinearity strength even without modifying the input Airy beam shape. This last feature makes this Airy soliton distinct from the Gaussian beam generated photorefractive soliton.

## Introduction

The Airy beam has been extensively studied in the last fifteen years since its first experimental observation in 2007^1^. The Airy beam originated from the domain of quantum mechanics in 1979 with the work of Berry and Balazs^2^ who found a shape-preserving accelerating solution to the potential-free Schrödinger equation in the form of an ideal Airy function. The paraxial wave equation is a mathematical equivalent of the Shrödinger potential-free equation, which allowed the same solution in optics. Due to the infinite tail of the Airy function and the impossibility to create infinitely large beams, the Airy beam profile is actually an exponentially truncated form of the analytic Airy function. Still, the truncated Airy beam shows characteristics of self-regeneration^3^, acceleration (or curved trajectory) and to a certain extent non-diffraction. The parabolic trajectory of the Airy beam has been studied^4^ as well as modified using dynamically varying linear index potentials^5^. Beams derived from the one-dimensional Airy beam are now being explored such as optical Airy bullets^6^, abruptly auto-focusing waves^7^, circular Airy beams^8^ or super-Airy beams^9^. These properties have led to a number of interesting applications such as curved plasma channel generations^10^, light-sheet microscopy^11^, particle manipulation^12^, plasmons^13^, material processing^14^ or all-optical routing^15–17^.

Airy beam propagation in nonlinear media has suggested interesting dynamics such as soliton-like behaviors and interactions of co- and counter-propagating Airy beams^18–22^. As a reminder, self-trapping of light beams has been originally observed by compensating the natural diffraction of light by using the focusing effect of nonlinear media^23,24^. Theoretically, the generation of stable 2D $\chi$ $^2$ solitons using a nonlinear frequency conversion phenomenon from Airy waves has been predicted in^25^; following this study, in similar quadratic optical media, the dynamics and interactions of 2D Airy waves and solitons emerging from a frequency conversion process has also been studied^26^. By using saturable nonlinear media such as photorefractive crystals, two dimensional self-trapping is also possible, meaning the beam self-traps in both axes transverse to the propagation axis^27^. In similar focusing nonlinear conditions the Airy beam may split into a weak accelerating structure and an “off-shooting soliton” (OSS) that propagates along the medium without transverse acceleration^21,28^. The analysis of the one-dimensional Airy beam propagation has proven that the OSS may behave as the expected photorefractive spatial soliton^22^. Still, there is so far neither theoretical and experimental evidence of a two-dimensional soliton emerging from an Airy beam nor any experimental study of two dimensional Airy beam propagating in a self focusing nonlinear medium. The understanding of such dynamics are of particular interest for the engineering of all-optical waveguides^16,17^.

In this paper we study experimentally the propagation of a 2D Airy beam inside a photorefractive strontium barium niobate (SBN) crystal for different values of light power and bias electric field applied on the crystal. We demonstrate the formation of a two dimensional Airy beam soliton. Several scenarios are identified showing the transition from the Airy beam to the soliton when increasing the nonlinearity strength of the medium. We show that the soliton output profile behaves according to the theoretical soliton existence curve and that it can be tailored through the nonlinearity strength even without modifying the input Airy beam shape. Hence, this feature distinguishes the Airy beam soliton from the Gaussian beam generated photorefractive soliton. In addition, the Airy soliton is formed at a distinct transverse coordinate than the expected linear Airy output, and this transverse splitting is interesting for the soliton analysis and its applications.

## Two dimensional Airy soliton

**Figure 1:**
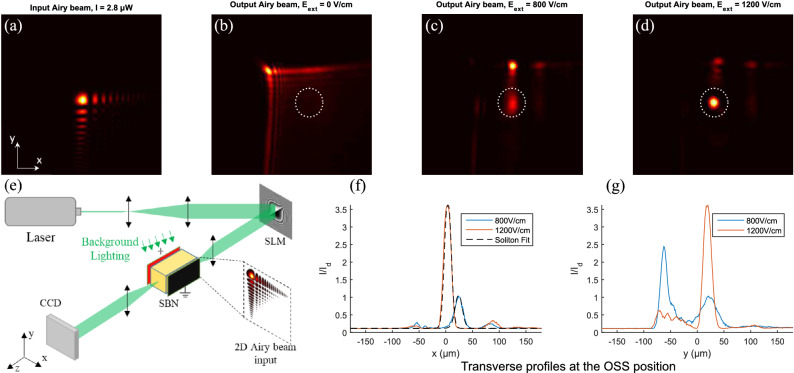
(**a**) Transverse power profile of the input Airy beam, $2.8\ \mu$W. (**b**–**d**) Transverse power profile of the output for different applied external field, $E_{ext}=0$ V/cm, $E_{ext}= 800$ V/cm, and $E_{ext}=1200$ V/cm, the soliton position is represented by a dotted white circle. (**e**) Schematic of the experimental setup. (**f**–**g**) power profiles along the x and y axis at the soliton coordinates, centered at the dotted white circle position.

The Airy beam profile injected inside the crystal (Fig. 1a) is obtained by modulating the phase of a Gaussian beam using a spatial light modulator (SLM). The SLM displays a 2D quadratic phase superimposed with a diffraction grating. Figure 1e shows a schematic of our experiment. We propagate a 2D Airy beam, of wavelength $\lambda = 532$ nm, through a photorefractive Cerium doped (0.01%) SBN:61 crystal ($n_{SBN}=2.3$) with dimensions 5 mm * 20 mm * 5 mm along 100, 010, 001 axis. The c axis of the crystal is placed along one of the 5 mm edge along the tetragonal axis and perpendicular to the propagation direction (20 mm edge) : it permits to exploit the dominant electro-optic coefficient of our SBN sample via a linearly polarized incident laser beam along c. Figures 1b–d shows the output face of the crystal for different values of externally applied electric field. After propagation through the SBN crystal the Airy beam seen in Fig. 1b is slightly diffracted and shifted due to the acceleration of the Airy beam. The Airy beam’s truncation parameter is chosen in function of the crystal’s length: it must be weak enough to preserve the Airy beam characteristics across the crystal, but strong enough to enable the observation of nonlinear effects on the beam’s propagation after a 2 cm propagation. When an external electric field is applied (Fig. 1c–d), the light focuses into a soliton-like beam that presents almost no shift in position when compared to the input Airy beam’s main lobe position (see the white circle). The dynamic of spatial light distribution in a photorefractive media is due to the interaction of light and matter and can be explained using the Kukhtarev band transport model^29^. The light propagation affects the refractive index variations and vice versa. This dynamic results in a focusing effect that, if sufficiently strong (Fig. 1c–d), modifies the profile of the propagating beam into a soliton.

When increasing the external field, the transition is gradual from Airy beam to soliton at the output face of the crystal. However we can distinguish two steps. The first step is the focusing along the preferential c-axis which is collinear to the applied external field and to the x-axis. Figure 1f (respectively (g)) shows the horizontal (respectively vertical) transverse output power profiles for applied fields of 800 V/cm and 1200 V/cm displayed in Fig. 1c,d. In Fig. 1c–d the light has focused horizontally along the x-axis to form an Airy soliton (Fig. 1f). This soliton is similar to the one-dimensional Airy soliton that would be induced by a one dimensional Airy beam^22^. However, with two dimensional Airy beams, a one-dimensional Airy beam structure is obtained along the vertical y-axis and competes with the Airy soliton (Fig. 1g). The second step occurs when focusing also takes place along the vertical y-axis, and this requires a stronger nonlinearity. Indeed, when further increasing the applied electric field, the light previously confined in the Airy beam structure transfers into a soliton-like structure as can be seen in Fig. 1g, and ultimately leads to the two dimensional Airy soliton. The process appearing in two steps will be explained in more details in the section entitled discussion and soliton analysis. To sum up, analysing the Airy beam focusing profile versus the applied electric field, different focusing strengths and soliton formation steps in x and y-axis are observed : this can be explained by the anisotropy of the crystal and the asymmetry of the electric field.

In order to compare the Airy beam soliton profile to the theoretical soliton profile, we test the theory developed for a one-dimensional steady state bright screening soliton profile which is described by the following reduced wave equation^30^:

1 $$\begin{aligned} \frac{d ^2 u}{d \xi ^2} + \frac{u}{u_0^2} \ln (1+u_0^2) - \frac{u}{1+u^2} = 0 \end{aligned}$$

whose first integral is

2 $$\begin{aligned} \frac{du}{d\xi }=[\ln (1+u^2)-(u^2/u_0^2)\ln (1+u_0^2 ) ]^{1/2} \end{aligned}$$

and where $u(\xi )$ is the soliton amplitude divided by the square root of the effective background intensity (defined as the sum of the background and dark intensities induced respectively by an external homogeneous illumination of the crystal and the intrinsic thermal excitation of charges inside the crystal), $\xi =x/d$ is the transverse coordinate normalized by $d=(k^2n_b^2r_{eff}E_{ext})^{-1/2}$, $u_0$ is the maximum amplitude of the soliton at $\xi =0$, $k=2\pi n_b/\lambda$ is the wave vector, $n_b$ is the unperturbed refractive index, $\lambda$ is the wavelength, $r_{eff}$ is the effective component of the electro-optic tensor, $E_{ext}=V/l$ with *V* the voltage applied onto the crystal and *l* the crystal’s width. As detailed in^22^, Eq. (2) is solved numerically using a Runge-Kutta method for an electric field applied along the x-axis and therefore gives the one-dimensional theoretical soliton profile along *x*. In practice, for (i) a different applied electric field $E_{ext}$ corresponding to a different *d* coefficient and (ii) a maximum soliton amplitude $u_0$, a different soliton profile is obtained. When we fix *d* and $u_0$ in our experiment ($n_b=2.3$, $r_{eff}=235\,$pm/V and $E_{ext}=800$ or 1200 V/cm), we therefore calculate the corresponding theoretical soliton profile. We then compare the superimposed soliton profile plots to the experimental profiles observed at the output face of our crystal in Fig. 1f, and both horizontal profiles along $x-axis$ fit perfectly. The profile along the vertical axis in Fig. 1g is slightly broader due to the elliptic shape of the soliton^31^.

## Tailoring the Airy soliton shape

**Figure 2:**
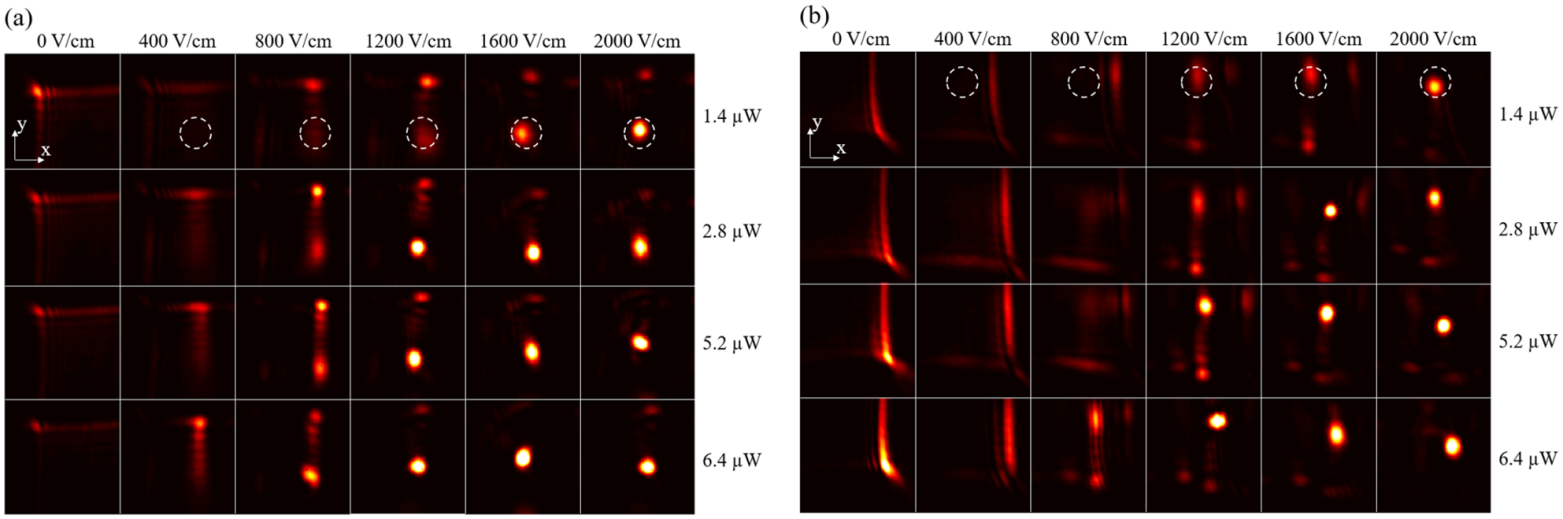
Beam profiles at the output of a 2 cm SBN crystal for input power values $I_{input}=1.4$–$6.4 \ \upmu$W and applied external field $E_{ext}=0$–2000 V/cm. (**a**) Airy beam of north-west acceleration. (**b**) Airy beam of south-east acceleration.

In order to show the effect of nonlinear strength on propagation behavior, Fig. 2 shows a panel of different output profiles after propagating through the SBN-crystal for different input powers and externally applied electric fields. The input power ranges from $1.4\,\upmu \hbox {W}$ to $6.4\,\upmu \hbox {W}$, the background lighting is constant at 35 mW and the applied electric field ranges from 0 V to 2000 V/cm. Figure 2a shows profiles for an input Airy beam with an acceleration direction along the negative x-axis and positive y-axis whereas for Fig. 2b the profile of the input Airy beam has a positive x-axis and negative y-axis acceleration direction. The same exposure time and attenuation in front of the camera are used for images belonging to the same row, but different attenuation and exposure time are used in the different rows. The image is taken once the Airy soliton is formed or when the output has stabilized. The soliton position is circled in white for the first row (light power of $1.4\,\upmu$W) for both Fig. 2a,b.

In Fig. 2a, when no electric field is applied, the 2D-Airy beam profile is unchanged. When an electric field of 400 V/cm is applied, the profile resembles a vertical 1D-Airy beam profile at the position in x where the soliton will form. Additionally, we notice the 1D-Airy beam profile appears more clearly at higher power ($6.4\,\upmu$W). When the applied electric field increases to 800 V/cm the residual vertical 1D-Airy beam profile is in competition with the soliton: for low power values ($1.4\,\upmu$W) the profile resembles a vertical 1D-Airy beam, whereas for higher power values ($6.4\,\upmu$W), both the 1D-Airy beam and the soliton are clearly discerned. When an electric field of 1200 V/cm is applied the 1D-Airy beam is still in competition with the soliton: for a beam power of $1.4\,\upmu$W both the 1D-Airy beam and soliton are discerned, whereas for a beam power of $6.4\,\upmu$W the 1D-Airy profile has almost disappeared to the benefit of the soliton. When an electric field of 1600 V/cm or 2000 V/cm is applied the soliton is very intense and other few off-shooting beams (OSB) of low power are discerned.

In Fig. 2b, when no electric field is applied, the 2D-Airy beam profile has already changed in comparison to the linear Airy beam propagation: all of the power has shifted to the main lobe along the x-axis. The asymmetry between Fig. 2a,b is to be expected: as explained in reference^32^, diffusion of carriers along the c-axis causes the light power to shift to the Airy beam’s main lobe. When an electric field of 400 V/cm is applied, the light focuses more strongly in the Airy beam’s main lobe. When an electric field of 800 V/cm is applied, light power starts to shift from the Airy beam’s main lobe position in *x* to the soliton position. When light shifts to the position of the soliton position in *x*, for $6.4\,\upmu$W, a residual vertical 1D-Airy beam is in competition with the soliton. When an electric field of 1200 V/cm is applied the light in the Airy beam’s main lobe focuses in an OSB and is in competition with the 1D-Airy beam and the soliton: for a beam power of $1.4\,\upmu$W to $5.5\,\upmu$W, three structure can be observed (Airy and soliton to the left, OSB to the right), whereas for a beam power of $6.4\,\upmu$W the OSB is no longer visible. When an electric field of 1600 V/cm is applied the Airy beam, soliton and OSB are still in competition: the three structures are discerned for light powers of $1.4\,\upmu$W to $2.8\,\upmu$W and only the soliton is very intense for light powers of $5.5\,\upmu$W and $6.4\,\upmu$W. When an electric field of 2000 V/cm is applied only the soliton remains. When comparing Fig. 2a,b, the differences in light propagation behavior occur mainly at low applied electric field when diffusion is a significant effect, but the behaviors become similar when a higher electric field is applied (greater or equal to 1200 V/cm).

The nonlinear effect can be increased by increasing the applied electric field and/or the light power injected in the crystal. In both Fig. 2a,b, increasing the nonlinear effect yields similar behaviors. The Airy beam profile disappears, replaced by a focused and centered soliton. Indeed, for a given injected light power and when increasing the applied external field, the output light power when propagating is focused first horizontally from 400 to 1200 V/cm and secondly is focused both horizontally and vertically from 1200 to 2000 V/cm. Similarly for a given externally applied electric field, for example 1200 V/cm, increasing the light power from 1.4 to $6.4\,\upmu$W allows focusing previously only along the x-axis to be focusing along both x and y axes. The c-axis of the SBN-crystal and the applied external field are both along the x-axis, explaining the stronger horizontal focusing effect and a two step focusing dynamic. Figure 2a,b present slightly different behaviors that can be due to different factors. The main identified factor is the diffusion effect as it is unidirectional and along the horizontal c-axis. It can be observed at 0 V/cm and the resulting profiles coincide with what can be expected in literature^32^.

When no voltage is applied in Fig. 2a the light shifts along the x-axis towards the soliton position, and in Fig. 2b the light distribution shifts along the x-axis to the position of the Airy beam’s main lobe. When the externally applied field is turned on an additional drift effect must be added to the diffusion effect. The shift increases with the value of the applied external field and light power, and causes the soliton to shift position at the output of the crystal or “bend” similarly to previous works with Gaussian solitons^33^. The bending effect is not apparent in Fig. 2 because the bending occurs after the soliton is formed, on a time scale ranging from a few dozen seconds to a few minutes. Time measurements show that the bending is faster when the light power or externally applied field increases.

## Discussion and soliton analysis

Regarding the soliton formation theory and the related nonlinear propagation equation, a 1D or 2D soliton generation needs a perfect balance between a nonlinear focusing process and the natural diffraction appearing when light propagates inside a medium^34,35^. Thanks to the photorefractive effect, focusing processes may occur when the travelling light induces by itself gradients of charges that redistribute themselves due to drift and/or diffusion effect in ferroelectric medium such as SBN crystals. As such, self bending of the resulting soliton can also be observed^33^. Furthermore, in 1D, self-localized beams can nevertheless be observed thanks to carrier diffusion effects and a smart tailoring of the accelerating component of the Airy structure^32^. In order to annihilate the bending trajectory of such a self-focused Airy beam, an external electric field applied along the appropriate direction (ferroelectric c-axis of the crystal) is necessary. Nevertheless, the SBN crystal electro-optic tensor, its anisotropy and the asymmetry of the applied electric field lead to an asymmetry in the charges redistribution in x (c-axis) and perpendicular y axis. As already explained in part II, this results in different focusing strengths and soliton formation along the x and y transverse axis explaining how the focusing behaviors in Figs. 1 and 2 appear in two steps. The horizontal c-axis, along which the voltage is applied, focuses more strongly than the vertical axis. Due to the saturation nature of the photorefractive effect, the focusing will saturate for both transverse axis but at different values of the applied electric fields. Before saturation is reached for both axis the beam is elliptic, once saturation is reached along both axis the soliton is circular. However, due to the strong applied electric field needed for saturation to be reached, the soliton also tends to self-bend in the direction of the applied electric field.

**Figure 3:**
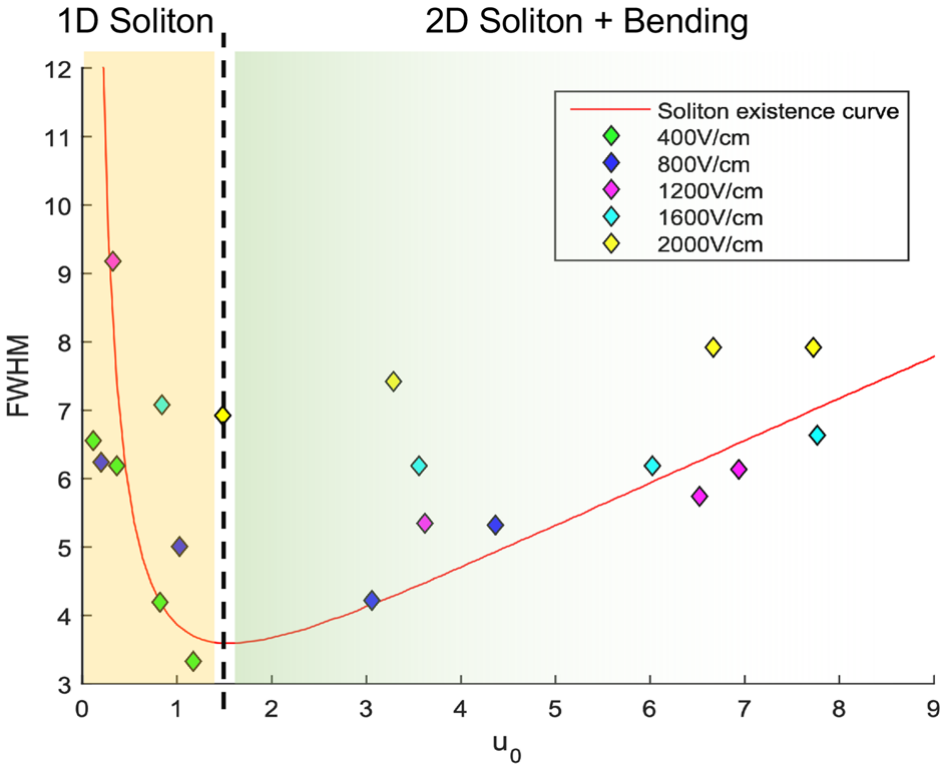
Theoretical soliton existence curve in red and experimental measures corresponding to Fig. 2a.

To further understand the dynamic of the Airy beam soliton behavior we can look at the soliton profile along the c-axis (x-axis) by plotting the full width at half maximum (FWHM) as a function of the maximum amplitude and superimposing it to the theoretical soliton existence curve. Indeed, equation (2) is solved numerically for different values of $u_{0}$. The FWHM of the amplitude profile $u(\xi )$ as a function of $u_0$ is represented in Fig. 3 in red. The experimental horizontal FWHM of the Airy soliton and the corresponding max amplitude from Fig. 2a are plotted as diamonds in Fig. 3. The experimental measures are scattered along the existence curve represented in red, and follow the same trend as the soliton existence curve for $u_0<1.5$ (orange area on Fig. 3) confirming the solitonic nature of the 1D observed focused beam (as predicted in^22^). Whereas the existence curve concerns only the 1D soliton theory, this curve is a good indicator of the expected Airy beam soliton propagation behavior: indeed, experimental measures corresponding to $u_0<1.5$ are for unidimensional horizontal focusing in agreement with the one-dimensional soliton theory, whereas experimental values corresponding to $u_0>1.5$ are for two dimensional focusing (green area on Fig. 3). Above $u_0>1.5$, the experimental values corresponding to the measured profiles for external applied fields $E_{ext}$ from 400V to 1200 V/cm (green, blue and pink diamonds in Fig. 3) follow the curve trend and starts slightly diverging from the fundamental curve for $E_{ext}$ equal to 1600 V/cm to 2000 V/cm (light blue and yellow diamonds in Fig. 3). The ones for $u_0>3$ lead to overfocusing or strong drift effects after a dozen seconds, showing less stable Off Shooting Solitons. Indeed, for high nonlinearities ($E_{ext}>1500$ V/cm or $u_0>3$), solitons are no more steady-state because of overfocusing phenomena accompanied by bending effect: we are in presence of quasi-steady or transient soliton. The different scenarii are illustrated in Fig. 3 where we represent the different steps in the observation of the solitonic behaviours: stable 1D and 2D solitons and quasi steady overfocused and drifted beams (orange and green areas on Fig. 3).

## Conclusions

In summary we have performed an in-depth experimental analysis of a two-dimensional Airy beam inside a photorefractive nonlinear medium. The nonlinearity strength can be changed by modifying the externally applied electric field and/or the Airy beam’s power. With weak nonlinearity, the two-dimensional input Airy beam turns into a one-dimensional output Airy beam. With moderate nonlinearity, the one dimensional Airy beam coexists with the soliton. With high nonlinearity the two-dimensional Airy beam converts completely into a two dimensional soliton. The soliton output profile behaves according to the theoretical soliton existence curve. When thinking about all-optical interconnects using soliton interactions, these different light propagation behaviors can be seen as different waveguiding configurations. The different configurations and soliton shape are obtained for the same Airy beam profile contrarily to Gaussian generated solitons that require the Gaussian beam to have an initial profile very close to the expected theoretical soliton profile.

## Data Availability

The datasets used and/or analysed during the current study are available from the corresponding author on reasonable request.
